# β-Cell Dysfunction and Altered Thyroid Hormone Dynamics in Post-COVID Metabolic Disturbances: An Immunometabolic Cross-Sectional Study

**DOI:** 10.3390/biomedicines14071420

**Published:** 2026-06-23

**Authors:** Victoria Tsvetkova, Malvina Todorova, Milena Atanasova, Irena Gencheva, Katya Todorova

**Affiliations:** 1 Clinic of Endocrinology, University Hospital “Georgi Stranski”—Pleven, 5800 Pleven, Bulgaria; 2 Department of Cardiology, Pulmonology and Endocrinology, Medical University—Pleven, 5800 Pleven, Bulgaria; 3 Department of Anatomy, Histology, Cytology and Biology, Biology Division, Medical University—Pleven, 5800 Pleven, Bulgaria; 4 Department of Clinical Immunology, Allergology and Clinical Laboratory, Clinical Laboratory Division, Medical University—Pleven, 5800 Pleven, Bulgaria

**Keywords:** COVID-19, post-COVID syndrome, β-cell dysfunction, insulin resistance, FT3, euthyroid sick syndrome, thyroid dysfunction, immunometabolism, metabolic syndrome

## Abstract

**Background**: SARS-CoV-2 infection has been associated with metabolic disturbances and endocrine alterations, including effects on pancreatic β-cell function and thyroid hormone regulation. However, the relationship between thyroid function and β-cell compensatory capacity in the post-COVID state remains unclear. **Methods**: In this cross-sectional study, we evaluated β-cell compensation (HOMA-B/HOMA-IR) and thyroid parameters in three groups: patients with active COVID-19, individuals with post-COVID metabolic disturbances, and a COVID-negative metabolic syndrome reference group. Thyroid status was assessed using both comprehensive clinical classification and biochemical criteria. Associations between thyroid hormones and β-cell function were analyzed using Spearman correlation. **Results:** β-cell compensatory capacity differed significantly across groups, with the lowest values observed during active COVID-19 and intermediate impairment in the post-COVID cohort compared with the metabolic syndrome group. FT3 concentrations and the FT3/FT4 ratio were significantly reduced during active infection and were positively associated with β-cell compensation in the post-COVID group (ρ = 0.421, *p* = 0.018 and ρ = 0.382, *p* = 0.031, respectively). Although thyroid dysfunction appeared more prevalent in the post-COVID cohort when defined by overall clinical classification, no significant differences were observed when thyroid status was evaluated based solely on biochemical criteria, excluding clinical history and euthyroid sick syndrome. **Conclusions:** Post-COVID metabolic disturbances are characterized by impaired β-cell compensatory capacity and alterations in peripheral thyroid hormone dynamics. The apparent increase in thyroid dysfunction is largely driven by pre-existing thyroid disease and non-thyroidal illness effects rather than intrinsic thyroid pathology. These findings are consistent with the hypothesis of a potential post-COVID immunometabolic phenotype involving both pancreatic and thyroid-related mechanisms.

## 1. Introduction

Since its emergence, coronavirus disease 2019 (COVID-19) has increasingly been recognized as a systemic disorder affecting multiple endocrine and metabolic pathways. Beyond the acute respiratory illness, accumulating evidence indicates that SARS-CoV-2 infection may lead to persistent metabolic disturbances, including impaired glucose regulation, insulin resistance (IR), and newly diagnosed diabetes or prediabetes. Large epidemiological studies have demonstrated an increased incidence of metabolic dysfunction following COVID-19, suggesting that the infection may trigger long-lasting alterations in metabolic homeostasis [1,2].

Pancreatic β-cell function plays a central role in maintaining glucose homeostasis by adjusting insulin secretion relative to peripheral insulin sensitivity. Disruption of this compensatory mechanism can lead to dysglycaemia and metabolic decompensation. Several mechanisms have been proposed to explain β-cell dysfunction in COVID-19, including inflammatory signaling, metabolic stress, and potential direct viral effects on pancreatic endocrine tissue [3,4]. However, clinical observations suggest that metabolic disturbances observed after COVID-19 do not fully align with classical metabolic syndrome (MetS), but instead reflect a distinct and heterogeneous metabolic phenotype [2,5].

The thyroid axis is a key regulator of systemic metabolism and energy balance. Thyroid hormones influence pancreatic β-cell activity, insulin secretion, and mitochondrial function, which are essential for glucose-stimulated insulin release [6,7,8,9]. Alterations in thyroid hormone metabolism are commonly observed during severe systemic illness, most notably a reduction in circulating free triiodothyronine (FT3), a phenomenon described as non-thyroidal illness syndrome (NTIS) also referred to as euthyroid sick syndrome (ESS) or low T3 syndrome [10].

During COVID-19 infection, several studies have reported abnormalities in thyroid function, including transient thyrotoxicosis, thyroiditis, and alterations in circulating thyroid hormone levels that correlate with disease severity and inflammatory activity [11,12,13]. In particular, reductions in circulating FT3 levels consistent with non-thyroidal illness physiology have been described in hospitalized patients with COVID-19 and have been associated with disease severity and inflammatory burden [14,15]. Moreover, emerging evidence suggests that thyroid axis alterations may persist beyond the acute phase of infection, with thyroid dysfunction reported during post-COVID follow-up in a subset of patients [16].

Despite growing interest in the endocrine consequences of COVID-19, the interaction between thyroid hormone dynamics and metabolic regulation in post-COVID metabolic phenotypes remains insufficiently characterized, particularly in light of emerging evidence linking post-COVID states with persistent metabolic and endocrine alterations [2,16]. In particular, few studies have simultaneously examined thyroid hormone profiles, thyroid autoimmunity, and indices of β-cell compensation across different stages of COVID-19 and in comparison with classical MetS. A better understanding of these relationships may provide important insight into the endocrine and immunometabolic mechanisms underlying post-COVID metabolic disturbances.

Given the central role of thyroid hormones in glucose metabolism, mitochondrial function, and insulin secretion, alterations in thyroid hormone dynamics may represent an additional endocrine component contributing to post-COVID metabolic phenotypes [6,7,8].

To address this gap, we performed a cross-sectional characterization of metabolic and thyroid profiles across three clinically distinct groups: individuals with active COVID-19 infection, patients with post-COVID metabolic disturbances, and a COVID-negative reference population with MetS.

We hypothesized that post-COVID metabolic dysfunction represents an intermediate immunometabolic phenotype, consistent with emerging evidence that post-COVID metabolic alterations may not fully overlap with classical MetS [2], and is characterized by impaired β-cell compensation and distinct alterations of the thyroid axis.

To better characterize the metabolic and endocrine alterations associated with SARS-CoV-2 infection, we designed a three-group comparative framework reflecting distinct clinical and metabolic states. Specifically, the study includes (i) patients with active COVID-19, representing acute infection-associated metabolic disturbance; (ii) individuals with post-COVID metabolic alterations, reflecting a post-infectious metabolic phenotype; and (iii) COVID-19-negative individuals with MetS, serving as a model of classical metabolic dysfunction unrelated to SARS-CoV-2 infection. This design allows differentiation between transient effects of acute illness and persistent metabolic alterations following COVID-19, as well as comparison with established metabolic disease.

Specifically, we aimed to determine whether thyroid hormone dynamics—particularly circulating FT3 levels and indices of peripheral thyroid hormone conversion—are associated with β-cell functional capacity and metabolic compensation in the post-COVID state.

## 2. Materials and Methods

### 2.1. Study Aim and Design

This was a prospective observational study with a cross-sectional analysis and aimed to investigate the relationship between thyroid axis alterations and pancreatic β-cell function across three clinical and metabolic states: active COVID-19 infection, post-COVID metabolic disturbances, and COVID-19-negative individuals with MetS. Participants were prospectively enrolled, and cross-sectional comparisons were performed between the study groups.

The study design allowed direct comparison of thyroid hormone dynamics, thyroid autoimmunity, and indices of β-cell compensation across acute infectious, post-infectious, and chronic metabolic conditions. This approach was intended to explore potential immunometabolic interactions linking thyroid dysfunction with impaired β-cell function in the context of COVID-19 and MetS.

### 2.2. Study Population and Grouping

Participants were recruited between January 2022 and December 2023 at the University Hospital Georgi Stranski—Pleven, Bulgaria. Patients with active COVID-19 infection were admitted to the Clinic for Pulmonology for treatment of acute SARS-CoV-2 infection, while individuals in the post-COVID and MetS groups were hospitalized in the Clinic of Endocrinology and metabolism for metabolic evaluation.

Participants were consecutively recruited from the respective clinical units during the study period based on predefined eligibility criteria.

The sample size was determined by the number of eligible patients available during the recruitment period and reflects an exploratory analysis of endocrine and metabolic alterations following COVID-19. Each group included more than 30 participants, enabling exploratory statistical comparisons.

A total of 100 adult participants were included (34 men and 66 women) and allocated into three groups according to SARS-CoV-2 infection status and metabolic characteristics.

**Group 1: Active COVID-19 (n = 32)**

This group included adults hospitalized with laboratory-confirmed SARS-CoV-2 infection, defined by a positive polymerase chain reaction (PCR) test at admission. Blood sampling was performed during hospitalization within the acute phase of infection (7–14 days from symptom onset).

Blood samples were collected prior to the initiation of pharmacological treatment, including antibiotic therapy, systemic glucocorticoids, or other COVID-19-related medications, in order to capture biochemical parameters during the early phase of SARS-CoV-2 infection.

**Group 2: Post-COVID cohort (n = 35)**

This group comprised individuals with newly diagnosed disturbances of carbohydrate metabolism identified at least 6 months after recovery from PCR-confirmed COVID-19 infection. This interval was selected to ensure evaluation during the post-acute phase of infection. The median interval between the acute SARS-CoV-2 infection and study enrolment was 7 months (Q1–Q3: 6–9 months).

All participants in this group had:

- Documented normoglycaemia prior to SARS-CoV-2 infection;
- No history of antidiabetic or metabolic treatment before COVID-19.

Disturbances of carbohydrate metabolism were defined according to World Health Organization and American Diabetes Association criteria [17,18] and included newly diagnosed impaired fasting glucose (IFG), impaired glucose tolerance (IGT), or diabetes mellitus (DM), including type 1 diabetes mellitus/latent autoimmune diabetes in adults (T1DM/LADA) and type 2 diabetes mellitus (T2DM).

Cases classified as T1DM or LADA in the post-COVID group represent newly diagnosed cases identified during the study evaluation. None of these participants had received insulin therapy prior to enrolment, reflecting the early stage of disease at diagnosis.

In addition, some participants demonstrated normoglycaemia with evidence of IR and/or hyperinsulinaemia during the oral glucose tolerance test (OGTT), characterized by disproportionately elevated insulin responses relative to plasma glucose concentrations during endocrine evaluation.

Participants were hospitalized in the Clinic of Endocrinology for detailed metabolic evaluation after abnormalities in glucose homeostasis were identified during routine follow-up following COVID-19 infection.

**Group 3: COVID-19-negative individuals with MetS (n = 33)**

This group comprised individuals with MetS, defined according to the 2009 harmonized criteria [19], without evidence of prior SARS-CoV-2 infection.

Absence of prior COVID-19 was defined by:

- Negative PCR testing;
- Absence of clinical and documented history suggestive of previous SARS-CoV-2 infection; serological testing was additionally considered when available.

MetS was defined by the presence of at least three of the following components:

- Abdominal obesity;
- Elevated triglycerides (TG);
- Reduced high-density lipoprotein cholesterol (HDL-C);
- Hypertension;
- Dysglycaemia.

This group was included to represent a reference model of established metabolic dysfunction unrelated to SARS-CoV-2 infection, allowing comparison with COVID-related metabolic alterations.

Disturbances in glucose homeostasis—including T2DM, prediabetes (IFG and IGT), or IR with normoglycaemia—were identified during the study evaluation and included both newly diagnosed and previously recognized cases.

Vaccination status

All participants across the three groups were unvaccinated against COVID-19 prior to enrolment.

The grouping strategy was designed to reflect clinically distinct metabolic contexts rather than matched populations. In particular, the post-COVID group was defined by newly developed metabolic disturbances following SARS-CoV-2 infection, which required documented normoglycaemia prior to infection. This criterion was not applicable to the other groups, which served as reference populations for acute infection and classical metabolic dysfunction. The MetS group was specifically included as a model of established, non–COVID-related metabolic disease, allowing comparison with post-COVID metabolic alterations. Therefore, comparisons between groups should be interpreted within the context of this exploratory design.

#### Assessment of COVID-19 Disease Severity

The severity of SARS-CoV-2 infection was assessed using a clinical classification system in accordance with the recommendations of the National Health Commission of China [20].

**COVID-19 severity** was categorized into four clinical forms:

- Mild—Defined by mild clinical symptoms without radiological evidence of pneumonia.
- Moderate—Defined by the presence of fever and/or respiratory symptoms with radiological evidence of pneumonia.
- Severe—Defined by respiratory distress (respiratory rate > 30 breaths/min), oxygen saturation ≤ 93% at rest, and/or a ratio of arterial partial pressure of oxygen to fractional inspired oxygen (PaO_2_/FiO_2_) ≤ 300 mmHg.
- Critical—Defined by respiratory failure requiring mechanical ventilation, shock, non-pulmonary organ failure, and/or admission to an intensive care unit.

Among patients in the active COVID-19 group (Group 1; n = 32), disease severity was distributed as follows: 6 individuals (18.75%) had mild disease, 7 (21.88%) had moderate disease, 16 (50.0%) had severe disease, and 3 (9.38%) had critical disease. The mean duration of hospitalization in this group was 10.45 days, and the in-hospital mortality rate was 6.25% (2 cases).

Clinical management of hospitalized patients with active COVID-19 followed the standard treatment protocols in place during the study period and was guided by disease severity and individual patient characteristics [21]. Therapeutic management included antibacterial therapy when clinically indicated, low-dose systemic glucocorticoids, anticoagulation therapy, and supportive symptomatic treatment.

Systemic glucocorticoids were administered at standard anti-inflammatory doses in accordance with national clinical guidelines and institutional protocols. Typical regimens included methylprednisolone at a dose of 32 mg/day or dexamethasone at a dose of 6 mg/day (or equivalent corticosteroid doses), with treatment duration determined by clinical response and disease severity.

Importantly, blood sampling for biochemical, hormonal, and immunological assessment in patients with active COVID-19 was performed prior to the initiation of pharmacological therapy, including antibiotic treatment, systemic glucocorticoids, or anticoagulants, in order to minimize the potential influence of treatment on measured biomarkers.

In the post-COVID cohort (Group 2), six individuals (17.14%) required hospitalization during the acute phase of SARS-CoV-2 infection and received standard inpatient management, including symptomatic treatment and, where clinically indicated, antibacterial therapy and low-dose systemic glucocorticoids. None of these patients required admission to an intensive care unit or mechanical ventilation.

Information on COVID-19 treatment and clinical course was obtained from hospital medical records and patient documentation.

Disease severity was recorded for descriptive purposes and was not incorporated as a covariate in the primary statistical analyses due to limited subgroup sizes and the exploratory nature of the study.

### 2.3. Inclusion Criteria

Participants were eligible for inclusion if they met all of the following criteria:

- Age ≥ 18 years;
- Laboratory-confirmed SARS-CoV-2 infection by positive PCR test at hospitalization (Group 1);
- Documented SARS-CoV-2 infection confirmed by PCR testing with a minimum interval of 6 months after the acute phase (Group 2);
- Newly diagnosed carbohydrate metabolism disturbances following SARS-CoV-2 infection with documented normoglycaemia prior to COVID-19 (Group 2);
- Diagnosis of MetS according to the harmonized criteria, defined by the presence of at least three of five components (Group 3);
- Negative PCR test for SARS-CoV-2 and no anamnestic or documented evidence of prior COVID-19 infection (Group 3).

### 2.4. Exclusion Criteria

Participants were excluded if any of the following were present:

- Age < 18 years or >90 years;
- Pregnancy or breastfeeding;
- Pre-existing T1DM or T2DM prior to SARS-CoV-2 infection (Group 2);
- Prior vaccination against COVID-19 (all groups);
- Known autoimmune disease;
- Severe or decompensated cardiovascular, respiratory, gastrointestinal, renal, or active malignant disease;
- Treatment with biological agents, immunosuppressive therapy, or cytotoxic drugs within the preceding 12 months;
- Systemic glucocorticoid therapy during COVID-19 treatment at doses ≥0.5–1.0 mg/kg/day methylprednisolone (or equivalent) for more than 10 days;
- Chronic systemic glucocorticoid use.

### 2.5. Ethics Approval and Informed Consent

The study was conducted after obtaining approval from the Ethics Committee of the Medical University of Pleven (Protocol No. 72/5 June 2023) and in accordance with the ethical principles of the Declaration of Helsinki.

All participants provided written informed consent prior to enrolment, confirming their voluntary participation and consent for the publication of de-identified clinical data. Participants were informed about the study objectives, procedures, and their right to withdraw at any time without consequences.

### 2.6. Clinical and Metabolic Assessment

All participants underwent a standardized clinical and metabolic evaluation, including detailed medical history, anthropometric measurements, and laboratory investigations.

Body mass index (BMI) was calculated as body weight (kg) divided by height squared (m^2^). BMI assessment was not feasible in hospitalized patients with acute COVID-19 (Group 1) due to clinical instability and infection control restrictions. Consequently, anthropometric comparisons involving BMI were limited to participants in Groups 2 and 3.

Routine biochemical measurements included:

- Fasting plasma glucose (mmol/L);
- Glycated haemoglobin (HbA1c);
- Lipid profile (total cholesterol, TG, HDL-cholesterol, LDL-cholesterol);
- Serum uric acid.

These measurements were used to characterize metabolic status and to support the diagnosis of MetS in the reference group.

### 2.7. Laboratory Measurements

Fasting venous blood samples were collected under standardized conditions, centrifuged within 30 min of collection, aliquoted, and stored at −80 °C until batch analysis. Repeated freeze–thaw cycles were avoided, and samples were thawed only once prior to analysis.

Laboratory assessment included standard biochemical, hormonal, and immunological parameters. Biochemical measurements were performed using standard automated laboratory methods as part of routine clinical evaluation, while hormonal analyses included insulin and thyroid hormones (TSH, FT3, FT4). Immunological analyses included thyroid autoantibodies (TPOAb, TgAb) and circulating cytokines, as described in the corresponding sections below.

### 2.8. Oral Glucose Tolerance Test (OGTT)

An oral glucose tolerance test (OGTT) was performed in participants from Group 2 (post-COVID cohort) and Group 3 (MetS reference group) in order to assess glucose metabolism and insulin responses. The test was performed using a 75 g oral glucose load after an overnight fast of at least 8 h.

Plasma glucose and insulin concentrations were measured at baseline (0 min), 60 min, and 120 min following glucose administration.

OGTT was not performed in individuals with fasting plasma glucose ≥ 11.1 mmol/L or in participants with previously diagnosed DM in the MetS group, in whom the diagnosis of diabetes was already established.

The OGTT results were also used to identify abnormalities in glucose metabolism and to evaluate IR and/or hyperinsulinemia relative to glucose responses.

### 2.9. Assessment of β-Cell Function

Pancreatic β-cell function and IR were assessed using the homeostasis model assessment (HOMA) [22].

IR was estimated using HOMA-IR, calculated as:

HOMA-IR = (fasting insulin × fasting glucose)/22.5

β-cell secretory capacity was estimated using HOMA-B, calculated as:

HOMA-B = (20 × fasting insulin)/(fasting glucose − 3.5)

Fasting plasma glucose concentrations were expressed in mmol/L, and fasting insulin concentrations were expressed in mIU/mL for all HOMA calculations.

Serum insulin concentrations were determined using electrochemiluminescence immunoassays (ECLIA) with Elecsys^®^ Insulin reagents on a Cobas e automated immunoassay analyzer (Roche Diagnostics, Mannheim, Germany) in the hospital clinical laboratory. The reference range for fasting insulin in the clinical laboratory was 2.60–24.90 mIU/L.

To assess β-cell compensation relative to IR, the HOMA-B/HOMA-IR ratio was calculated.

Because no established clinical cutoffs exist for HOMA-B/HOMA-IR, a cohort-specific threshold was used. β-cell dysfunction was defined as HOMA-B/HOMA-IR < 34.8, corresponding to the 25th percentile of the COVID-19-negative MetS reference group (Group 3).

This percentile-based approach was used to identify individuals with relatively impaired β-cell compensatory capacity and is consistent with previously published approaches using cohort-specific thresholds when standardized clinical cutoffs are not available, particularly in exploratory clinical studies [22,23].

### 2.10. Thyroid Assessment and Definition of Thyroid Dysfunction

#### 2.10.1. Thyroid Hormone and Autoantibody Assessment

Thyroid function was evaluated by measurement of serum thyroid-stimulating hormone (TSH), free triiodothyronine (FT3), and free thyroxine (FT4), together with thyroid autoantibodies, including anti-thyroid peroxidase antibodies (TPOAb) and anti-thyroglobulin antibodies (TgAb/TAT).

Serum concentrations of TSH, FT3, and FT4 were determined using ECLIA on a Cobas e 411 automated immunoassay analyzer (Roche Diagnostics, Mannheim, Germany) in the hospital clinical laboratory.

Thyroid autoantibodies (TPOAb and TgAb) were measured using enzyme-linked immunosorbent assay (ELISA) methods according to the standard procedures of the clinical laboratory.

The reference ranges used in the laboratory were: TSH 0.20–4.20 mIU/L, FT3 3.10–6.80 pmol/L, FT4 11.9–21.6 pmol/L, TPOAb < 60 IU/mL, and TgAb < 60 IU/mL. Thyroid autoantibody positivity was defined as antibody concentrations exceeding the upper reference limit of the assay.

All laboratory analyses were performed in the same certified clinical laboratory using standardized procedures and internal quality control protocols.

The FT3/FT4 ratio was calculated as an indicator of peripheral thyroid hormone conversion.

#### 2.10.2. Thyroid Ultrasound Evaluation

Thyroid ultrasonography was performed in all participants from Groups 2 and 3 as part of the endocrine evaluation.

Ultrasound examination was used to assess thyroid morphology and to support the diagnosis of thyroid disorders. Particular attention was given to sonographic features suggestive of autoimmune thyroid disease, including diffuse parenchymal hypoechogenicity and heterogeneous echotexture.

Ultrasound findings were considered complementary to biochemical assessment in the classification of thyroid status.

#### 2.10.3. Thyroid Dysfunction Definition

Thyroid dysfunction was defined using a composite clinical definition that incorporated biochemical abnormalities, thyroid autoimmunity, and documented thyroid disease.

Participants were classified as having thyroid dysfunction if any of the following were present:

- Abnormal serum TSH, FT3, or FT4 levels outside the laboratory reference range;
- Positive thyroid autoantibodies (TPOAb or TgAb/TAT) in the presence of biochemical thyroid abnormalities;
- Documented thyroid disease and/or treatment with thyroid hormone replacement therapy.

Ultrasound findings suggestive of autoimmune thyroid disease (e.g., diffuse hypoechogenicity or heterogeneous parenchymal echotexture) were considered supportive diagnostic features, particularly in participants with negative thyroid autoantibodies.

For analytical purposes, thyroid status was evaluated both as an overall clinical classification and, separately, according to biochemical thyroid function categories.

Within the post-COVID cohort (Group 2), participants were further stratified into three thyroid status subgroups for exploratory analyses:

1. No thyroid dysfunction (No TD)—Normal thyroid hormone levels and no history of thyroid disease.
2. Pre-existing thyroid disease (PTD)—Thyroid disorder diagnosed prior to SARS-CoV-2 infection.
3. Newly detected thyroid dysfunction (NDTD)—No previous thyroid disease but abnormal thyroid function tests and/or positive thyroid autoantibodies identified during the post-COVID evaluation.

Newly detected thyroid dysfunction (NDTD) was defined based on a composite assessment, including not only biochemical abnormalities but also the presence of thyroid autoimmunity (positive TPOAb or TgAb) and/or ultrasound features suggestive of thyroid disease. As a result, some participants classified as NDTD may have had normal circulating thyroid hormone levels (biochemically euthyroid) but evidence of underlying thyroid pathology.

Levothyroxine (LT4) therapy was recorded based on patient medical history and medication review at the time of hospitalization.

#### 2.10.4. Definitions of Thyroid Function Categories

Based on the laboratory reference ranges, thyroid function status was classified as follows:

- Euthyroidism: TSH, FT3 and FT4 within the reference range.
- Subclinical hypothyroidism: TSH > 4.20 mIU/L with normal FT4.
- Overt hypothyroidism: TSH > 4.20 mIU/L with FT4 < 11.9 pmol/L.
- Subclinical hyperthyroidism: TSH < 0.20 mIU/L with normal FT4 and FT3.
- Overt hyperthyroidism: TSH < 0.20 mIU/L with FT4 > 21.6 pmol/L and/or FT3 > 6.80 pmol/L.
- Euthyroid sick syndrome (low T3 syndrome): FT3 < 3.10 pmol/L with normal TSH and FT4.

### 2.11. Cytokine Measurement

To explore potential immunometabolic mechanisms underlying thyroid alterations in the post-COVID cohort, circulating inflammatory cytokines were measured in a subset of Group 2 participants based on serum sample availability.

Circulating inflammatory cytokines were measured using commercially available enzyme-linked immunosorbent assay (ELISA) kits according to the manufacturers’ instructions (Elabscience, Houston, TX, USA).

The following cytokines were quantified:

- Tumor necrosis factor alpha (TNF-α)—Human TNF-α ELISA Kit (Elabscience, Houston, TX, USA). Assay sensitivity: 4.69 pg/mL. Detection range: 7.81–500 pg/mL.
- Interferon gamma (IFN-γ)—Human IFN-γ ELISA Kit (Elabscience, Houston, TX, USA). Assay sensitivity: 9.38 pg/mL. Detection range: 15.63–1000 pg/mL.
- Interleukin-17A (IL-17A)—Human IL-17A ELISA Kit (Elabscience, Houston, TX, USA). Assay sensitivity: 18.75 pg/mL. Detection range: 31.25–2000 pg/mL.
- Interleukin-10 (IL-10)—Human IL-10 ELISA Kit (Elabscience, Houston, TX, USA). Assay sensitivity: 0.94 pg/mL. Detection range: 1.56–100 pg/mL.
- Interleukin-7 (IL-7)—Human IL-7 ELISA Kit (Elabscience, Houston, TX, USA). Assay sensitivity: 4.69 pg/mL. Detection range: 7.81–500 pg/mL.

All ELISAs were performed according to the manufacturers’ protocols. All samples were analyzed in duplicate and concentrations were calculated from standard calibration curves.

Approximately 20 participants were included in the cytokine analysis, with the exact number varying by biomarker due to sample availability.

Because not all samples were available for all assays, analyses were conducted using available-case data, and the number of participants included in each analysis is reported in the corresponding tables.

### 2.12. Statistical Analysis

Statistical analyses were performed using IBM SPSS Statistics, version 25 (IBM Corp., Armonk, NY, USA).

Continuous variables are presented as mean ± standard deviation (SD) and median (Q1–Q3).

Normality of data distribution was assessed using the Shapiro–Wilk test. As at least one group for each variable demonstrated a non-normal distribution, non-parametric statistical methods were applied for primary group comparisons.

Comparisons among more than two groups were performed using the Kruskal–Wallis test, followed by Dunn’s post hoc test with Bonferroni correction when the omnibus test was significant. Two-group comparisons were conducted using the Mann–Whitney U test.

Categorical variables were analyzed using the chi-square (χ^2^) test.

Associations between continuous variables were evaluated using Spearman’s rank correlation coefficient (ρ) with corresponding 95% confidence intervals (CI).

To further evaluate group differences while accounting for potential demographic confounding, generalized linear models (GLM) were constructed using a gamma distribution with a log link function. Biomarker concentrations were included as dependent variables, study group was included as a categorical independent variable, and age and sex were included as covariates. Model results are presented as exponentiated regression coefficients (Exp(β)) with 95% confidence intervals (CI). A gamma distribution with a log link function was selected due to the positively skewed distribution of biomarker concentrations.

All statistical tests were two-sided, and a *p* value < 0.05 was considered statistically significant. For post hoc comparisons, Bonferroni-adjusted *p* values were used to determine statistical significance.

Analyses were performed using available-case data, and the number of participants included in each analysis is reported in the corresponding tables. This approach was particularly relevant for cytokine analyses, where measurements were available only in a subset of participants.

## 3. Results

### 3.1. Study Population Characteristics

A total of 100 participants were included in the study and categorized into three groups according to SARS-CoV-2 infection status and metabolic characteristics. The study population comprised 34 men (34%) and 66 women (66%).

Group 1 consisted of patients hospitalized with active COVID-19 infection (n = 32). Group 2 included post-COVID individuals evaluated at least 6 months after recovery from SARS-CoV-2 infection who presented with newly identified disturbances of carbohydrate metabolism (n = 35). Group 3 comprised COVID-19-negative individuals with MetS, serving as the reference population (n = 33).

Baseline demographic and clinical characteristics of the study population are presented in Table 1.

Significant differences in age were observed across the study groups, with participants in the active COVID-19 group being older than those in the post-COVID and MetS groups.

### 3.2. Glucose Metabolism Characteristics

The distribution of glucose metabolism disorders and antidiabetic therapy across the study groups is presented in Table 2.

Notably, cases classified as T1DM or LADA in the post-COVID group represent newly diagnosed patients identified during the study evaluation, and none had initiated insulin therapy at the time of assessment, reflecting the early stage of disease at diagnosis.

### 3.3. Metabolic and Thyroid Function Parameters

Baseline metabolic and thyroid function characteristics of the study population are presented in Table 3.

Overall, significant differences were observed across the three groups in key metabolic and thyroid parameters, indicating distinct clinical profiles.

Indices of β-cell function differed significantly across the study groups (Table 3). In addition, significant differences were observed in thyroid-related parameters, including TSH, FT3, FT4, and FT3/FT4 ratio. These findings are presented in detail in the following sections.

### 3.4. β-Cell Dysfunction Across Study Groups

Indices of β-cell compensatory capacity differed significantly across the three study groups (Table 3). The HOMA-B/HOMA-IR ratio showed a significant overall difference between groups (Kruskal–Wallis H(2) = 22.17, *p* < 0.001).

**Figure 1:**
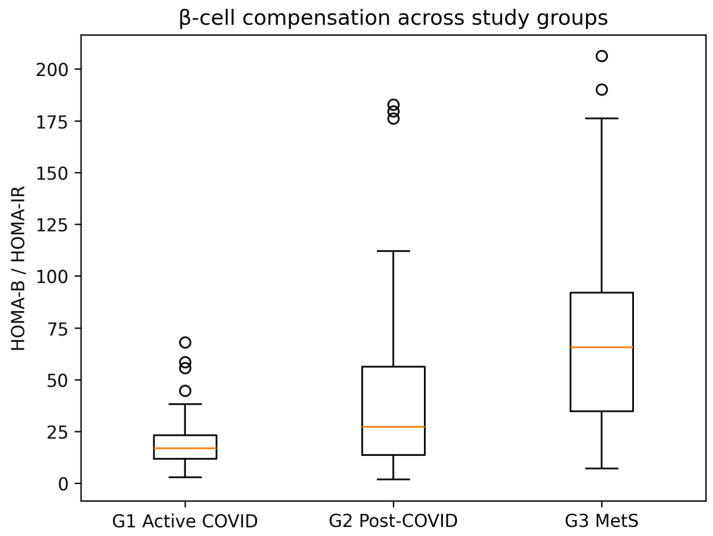
HOMA-B/HOMA-IR ratio across study groups. Box plots showing β-cell compensatory capacity in active COVID-19 (G1), post-COVID (G2), and COVID-negative MetS (G3) groups. Boxes represent the interquartile range, horizontal lines indicate the median, whiskers represent the range excluding outliers, and circles denote outliers.

Values were lowest in individuals with active COVID-19 and highest in the MetS reference group, while the post-COVID cohort demonstrated intermediate values (Figure 1).

Post hoc analysis using Dunn’s test with Bonferroni correction confirmed that HOMA-B/HOMA-IR values were significantly lower in the active COVID-19 group compared with both the post-COVID cohort (*p* < 0.001) and the MetS group (*p* < 0.001). In addition, the post-COVID group exhibited significantly lower values compared with the MetS group (*p* = 0.010).

Consistent with these findings, the prevalence of β-cell dysfunction differed significantly across the study groups (χ^2^(2) = 20.40, *p* < 0.001), with the highest proportion observed in patients with active COVID-19 and the lowest in the MetS group.

To further evaluate these differences while accounting for potential confounding, a generalized linear model (GLM) with gamma distribution and log link adjusted for age and sex was applied.

After adjustment, β-cell compensatory capacity (HOMA-B/HOMA-IR) remained significantly lower in individuals with active COVID-19 compared with the MetS group (Exp(β) = 0.29, 95% CI 0.19–0.44, *p* < 0.001). Similarly, the post-COVID cohort showed significantly lower HOMA-B/HOMA-IR values compared with the MetS group (Exp(β) = 0.62, 95% CI 0.43–0.88, *p* = 0.009). Direct comparison between the two COVID-related groups confirmed significantly lower β-cell compensation in active COVID-19 compared with post-COVID individuals (Exp(β) = 0.47, 95% CI 0.32–0.70, *p* < 0.001).

Detailed pairwise comparisons and full model results are presented in Supplementary Tables S1 and S2.

### 3.5. Thyroid Profile Across Study Groups

Thyroid hormone parameters differed significantly across the three study groups (Table 3).

Serum TSH concentrations varied significantly between groups (Kruskal–Wallis H(2) = 20.59, *p* < 0.001). Post hoc Dunn analysis demonstrated significantly lower TSH levels in patients with active COVID-19 compared with both the post-COVID cohort and the MetS group (both *p* < 0.001), while no significant difference was observed between the latter two groups.

A similar pattern was observed for FT3 concentrations (H(2) = 47.03, *p* < 0.001). FT3 levels were significantly reduced in individuals with active COVID-19 compared with both the post-COVID and MetS groups (both *p* < 0.001), with no significant difference between the post-COVID and MetS groups.

FT4 concentrations also differed significantly across the study groups (H(2) = 14.83, *p* < 0.001). Post hoc analysis revealed significantly lower FT4 levels in the post-COVID cohort compared with patients with active COVID-19 (*p* = 0.003), whereas other pairwise comparisons were not statistically significant.

The FT3/FT4 ratio demonstrated the most pronounced group differences (H(2) = 42.94, *p* < 0.001), with significantly lower ratios observed in patients with active COVID-19 compared with both the post-COVID and MetS groups (both *p* < 0.001).

In contrast, thyroid autoimmunity markers did not differ significantly across the study groups, including TPOAb (H(2) = 3.33, *p* = 0.190) and TgAb (H(2) = 0.54, *p* = 0.765).

Detailed pairwise comparisons from Dunn’s post hoc tests are presented in Supplementary Table S3.

To assess whether these differences persisted after adjustment for demographic covariates, GLM (gamma, log link) adjusted for age and sex was applied.

After adjustment, FT3 concentrations remained significantly lower in individuals with active COVID-19 compared with both the MetS group (Exp(β) = 0.65, 95% CI 0.56–0.74, *p* < 0.001) and the post-COVID cohort (Exp(β) = 0.68, 95% CI 0.60–0.78, *p* < 0.001). Similarly, TSH levels remained significantly lower in the active COVID-19 group compared with the MetS group (Exp(β) = 0.34, 95% CI 0.15–0.78, *p* = 0.011).

FT4 concentrations were significantly lower in the post-COVID cohort compared with the MetS group (Exp(β) = 0.87, 95% CI 0.79–0.96, *p* = 0.007). The FT3/FT4 ratio also remained significantly reduced in individuals with active COVID-19 compared with both the MetS group (Exp(β) = 0.65, *p* < 0.001) and the post-COVID cohort (Exp(β) = 0.59, *p* < 0.001).

No significant adjusted differences were observed for thyroid autoantibody levels across groups, with the exception of a significant difference in TPOAb levels between the active COVID-19 and post-COVID groups (Exp(β) = 0.28, 95% CI 0.09–0.82, *p* = 0.021).

Full model results are presented in Table 4.

### 3.6. Prevalence of Thyroid Dysfunction Across Study Groups

When thyroid dysfunction was defined according to the overall study classification—including biochemical abnormalities, thyroid autoimmunity, and documented clinical history of thyroid disease or treatment—its prevalence differed significantly across the study groups (χ^2^(2) = 9.86, *p* = 0.007). Thyroid dysfunction was observed in 21.9% of individuals with active COVID-19, 57.1% of post-COVID patients, and 53.1% of participants with MetS (Table 3).

Post hoc pairwise comparisons with Bonferroni correction demonstrated that thyroid dysfunction was significantly more frequent in the post-COVID cohort compared with patients with active COVID-19 (*p* = 0.007), whereas differences involving the MetS group were not statistically significant after correction.

The prevalence of newly diagnosed thyroid dysfunction and levothyroxine therapy did not differ significantly across groups.

By contrast, when thyroid status was evaluated based on biochemical thyroid function categories alone, excluding clinical history and euthyroid sick syndrome, the prevalence of current biochemical thyroid dysfunction was lower and did not differ significantly across groups (9.3%, 17.1%, and 12.1% in groups 1, 2, and 3, respectively; χ^2^(2) = 0.92, *p* = 0.63), indicating no clear between-group pattern.

The distribution of specific thyroid function categories is presented in Table 5.

Euthyroidism was most frequent in the MetS group, followed by the post-COVID group, and least frequent in the active COVID-19 group. Notably, euthyroid sick syndrome (ESS, low T3 syndrome) was observed exclusively in patients with active COVID-19 (43.8%) and was absent in the post-COVID and MetS groups.

Other thyroid function categories, including subclinical and overt hypothyroidism, were observed at low frequencies across all groups without a consistent between-group pattern.

When thyroid function was analyzed as a binary outcome (euthyroid vs. thyroid dysfunction), thyroid dysfunction was significantly more frequent in patients with active COVID-19 compared with the post-COVID and MetS groups (65.6% vs. 25.7% vs. 12.1%, χ^2^(2) = 24.7, *p* < 0.001). This difference was primarily driven by the inclusion of euthyroid sick syndrome in the composite definition, which was observed exclusively in patients with active COVID-19.

**Figure 2:**
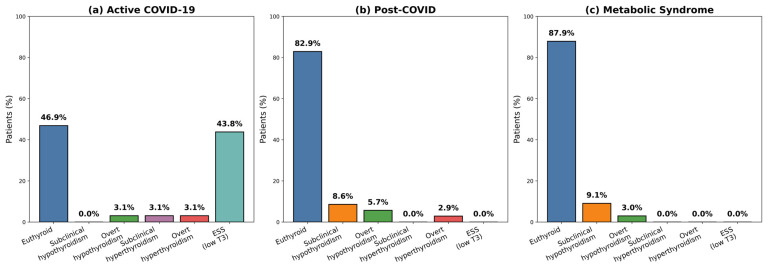
Distribution of thyroid function categories across study groups. Bar plots showing the percentage of patients with euthyroidism, subclinical hypothyroidism, overt hypothyroidism, subclinical hyperthyroidism, overt hyperthyroidism, and euthyroid sick syndrome (ESS; low T3 with normal TSH and FT4) in (**a**) active COVID-19, (**b**) post-COVID, and (**c**) COVID-negative MetS groups.

The distribution of thyroid function categories across study groups is illustrated in Figure 2.

### 3.7. Thyroid Status Subgroups Within the Post-COVID Cohort

Within the post-COVID cohort (Group 2), participants were stratified into three subgroups according to thyroid status: no thyroid dysfunction (No TD, n = 14), pre-existing thyroid disease (PTD, n = 9), and newly detected thyroid dysfunction (NDTD, n = 12).

The distribution of thyroid function categories across these subgroups is presented in Table 6. The apparent predominance of euthyroid status in the NDTD subgroup reflects the inclusion of participants with thyroid autoimmunity or ultrasound abnormalities in the absence of overt biochemical dysfunction.

Euthyroidism was most frequent in individuals without thyroid dysfunction and least frequent in those with pre-existing thyroid disease.

Comparisons of hormonal and immunological parameters between subgroups are shown in Table 7.

No statistically significant differences in the prevalence of β-cell dysfunction were observed across thyroid dysfunction subgroups.

Serum TSH levels differed significantly across the three subgroups (Kruskal–Wallis H(2) = 11.53, *p* = 0.003). Post hoc analysis demonstrated significantly higher TSH concentrations in individuals with pre-existing thyroid disease compared with those with newly detected thyroid dysfunction (adjusted *p* = 0.002), whereas other pairwise comparisons were not statistically significant.

Similarly, TPOAb levels differed significantly between subgroups (H(2) = 12.27, *p* = 0.002). TPOAb concentrations were significantly higher in both the pre-existing thyroid disease (*p* = 0.006) and newly detected thyroid dysfunction groups (*p* = 0.004) compared with individuals without thyroid dysfunction.

Although TAT levels differed across subgroups (H(2) = 6.19, *p* = 0.045), these differences did not remain significant after correction for multiple comparisons.

In contrast, no significant differences were observed between subgroups for FT3, FT4, the FT3/FT4 ratio, or indices of β-cell compensatory capacity, including HOMA-B/HOMA-IR.

Detailed pairwise comparisons are presented in Supplementary Table S4.

To further evaluate these associations, GLM (gamma, log link) adjusted for age, sex, and BMI were applied using the No TD subgroup as the reference category. In these models, TSH concentrations remained significantly higher in individuals with pre-existing thyroid disease compared with those with newly detected thyroid dysfunction (Exp(β) = 1.84, 95% CI 1.14–2.98, *p* = 0.013).

TPOAb levels remained markedly elevated in both the pre-existing thyroid disease (Exp(β) = 15.67, 95% CI 4.06–60.54, *p* < 0.001) and newly detected thyroid dysfunction groups (Exp(β) = 6.86, 95% CI 2.14–21.97, *p* = 0.001) compared with individuals without thyroid dysfunction. TgAb levels were also higher in participants with pre-existing thyroid disease (Exp(β) = 2.74, 95% CI 1.04–7.21, *p* = 0.041), while no significant difference was observed for newly detected thyroid dysfunction.

No significant adjusted differences were observed for FT3, FT4, FT3/FT4 ratio, or HOMA-B/HOMA-IR across subgroups.

Although not statistically significant, the highest proportion of β-cell dysfunction was observed in individuals with newly detected thyroid dysfunction.

Full model results are presented in Supplementary Table S5.

### 3.8. Correlations Between Thyroid Hormones and β-Cell Function in the Post-COVID Cohort

Within the post-COVID cohort, associations between thyroid hormone parameters and indices of β-cell function were assessed using Spearman rank correlation analysis (Supplementary Table S6).

FT3 concentrations demonstrated a moderate positive correlation with HOMA-B/HOMA-IR (ρ = 0.421, 95% CI 0.083–0.672, *p* = 0.018, n = 35), indicating that higher circulating FT3 levels were associated with greater β-cell compensatory capacity relative to IR. Similarly, the FT3/FT4 ratio was positively correlated with HOMA-B/HOMA-IR (ρ = 0.382, 95% CI 0.041–0.643, *p* = 0.031).

In contrast, TSH concentrations were not significantly associated with β-cell compensation (ρ = −0.092, 95% CI −0.418–0.263, *p* = 0.610). No significant correlations were observed for FT4 or thyroid autoantibodies (TPOAb and TgAb) with HOMA-B/HOMA-IR (all *p* > 0.05).

These findings suggest that peripheral thyroid hormone activity, as reflected by FT3 levels and the FT3/FT4 ratio, is associated with β-cell functional reserve in the post-COVID metabolic state.

**Figure 3:**
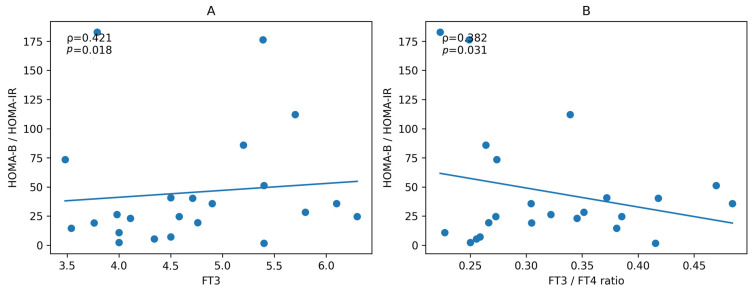
Associations between thyroid hormone indices and β-cell compensatory capacity in the post-COVID cohort. (**A**) Relationship between serum FT3 concentrations and HOMA-B/HOMA-IR. (**B**) Relationship between the FT3/FT4 ratio and HOMA-B/HOMA-IR. Blue circles represent individual participants, and the solid blue line shows the fitted linear regression trend for visualization purposes. Associations were assessed using Spearman rank correlation; corresponding Spearman correlation coefficients (ρ) and *p*-values are displayed in each panel.

The associations between FT3 and β-cell compensatory capacity, as well as between the FT3/FT4 ratio and HOMA-B/HOMA-IR, are illustrated in Figure 3.

### 3.9. Exploratory Cytokine Analysis

To explore potential immunometabolic heterogeneity within the post-COVID cohort, circulating cytokine concentrations were compared across thyroid status subgroups, including tumor necrosis factor-α (TNF-α), interferon-γ (IFN-γ), interleukin-17A (IL-17A), interleukin-10 (IL-10), and interleukin-7 (IL-7). The cytokine analysis was conducted as an exploratory component of the study to investigate potential immunological mechanisms underlying post-COVID metabolic alterations. Due to limited sample availability, these findings should be interpreted with caution.

Comparisons were performed using the Kruskal–Wallis test. No statistically significant differences in cytokine concentrations were observed between individuals with no thyroid dysfunction, pre-existing thyroid disease, and newly detected thyroid dysfunction (all *p* > 0.05).

Descriptive cytokine data for each subgroup are presented in Table 8.

## 4. Discussion

### 4.1. Principal Findings

The present study investigated the relationship between thyroid function and β-cell compensatory capacity across three clinically distinct metabolic states: active COVID-19 infection, post-COVID metabolic disturbance, and COVID-negative MetS.

Several key findings emerged. First, indices of β-cell compensation differed significantly across the three groups. The lowest HOMA-B/HOMA-IR values were observed during active COVID-19 infection, whereas the highest values were found in individuals with MetS. The post-COVID cohort demonstrated intermediate β-cell compensatory capacity compared with the other groups, which may indicate partial recovery but also potential persistence of metabolic alterations following SARS-CoV-2 infection.

Second, thyroid hormone parameters varied across study groups. Patients with active COVID-19 exhibited significantly lower FT3 concentrations and reduced FT3/FT4 ratios than both the post-COVID and MetS groups, consistent with altered peripheral thyroid hormone metabolism during acute systemic illness [10,11,12].

Third, thyroid dysfunction was more frequently observed in the post-COVID cohort when defined by the overall clinical classification. Subgroup analyses demonstrated heterogeneity in thyroid status, with significant differences in TSH and thyroid autoimmunity markers, particularly anti-thyroid peroxidase antibodies.

Fourth, within the post-COVID cohort, FT3 concentrations and the FT3/FT4 ratio were positively associated with HOMA-B/HOMA-IR, whereas TSH and thyroid autoantibodies were not. Exploratory cytokine analyses did not reveal significant differences across thyroid-status subgroups.

Taken together, these findings indicate an association between thyroid hormone alterations and impaired β-cell compensatory capacity across different clinical stages of COVID-19-related metabolic disturbance. These results should be interpreted in the context of a post-COVID cohort defined by newly developed metabolic abnormalities, rather than a general convalescent population.

Overall, the results are consistent with the concept of a potential post-COVID immunometabolic phenotype linking thyroid hormone dynamics with β-cell functional balance; however, this interpretation requires confirmation in larger and longitudinal studies.

### 4.2. β-Cell Dysfunction Across COVID-19 Stages

In the present study, indices of β-cell compensation differed markedly across the three metabolic states examined. The lowest HOMA-B/HOMA-IR values were observed in patients with active COVID-19, whereas the highest values were found in individuals with MetS without prior SARS-CoV-2 infection. Participants evaluated after recovery from COVID-19 demonstrated intermediate values compared with the other groups, which may reflect partial recovery but also possible persistence of metabolic alterations relative to IR.

These findings are consistent with accumulating evidence that SARS-CoV-2 infection has been proposed to influence glucose homeostasis through multiple mechanisms affecting pancreatic β-cell function. Experimental and clinical studies suggest that systemic inflammation, metabolic stress, and potential direct viral effects on pancreatic endocrine tissue may contribute to impaired insulin secretion and dysglycaemia during COVID-19 [3,4,24]. Clinical observations during the pandemic likewise reported increased rates of hyperglycemia and newly diagnosed diabetes among patients with COVID-19 [1].

The gradient observed in our study—from markedly reduced β-cell compensation during active infection, to intermediate values in the post-COVID cohort, to higher values in individuals with MetS—may indicate that metabolic alterations associated with COVID-19 differ in part from the classical pathophysiological pattern of MetS. In MetS, IR is often accompanied by compensatory hyperinsulinemia and relatively preserved β-cell secretion during earlier stages. By contrast, our findings suggest that acute COVID-19 may be associated with relatively lower β-cell compensatory capacity, potentially reflecting the combined effects of systemic illness, inflammatory signaling, and metabolic stress on pancreatic endocrine function [3,24].

The persistence of intermediate β-cell compensation in the post-COVID cohort is particularly notable. Importantly, this group comprised individuals with newly identified disturbances of carbohydrate metabolism following SARS-CoV-2 infection, rather than a general post-COVID population. Despite evaluation several months after recovery from acute infection, β-cell functional indices in this cohort remained significantly lower than those observed in the MetS reference group.

This finding may indicate that the observed impairment in β-cell compensatory capacity is not limited to the acute phase of illness and could persist beyond recovery, although this cannot be definitively established in the present cross-sectional design. This observation is consistent with population-based evidence demonstrating an increased risk of metabolic disorders and new-onset diabetes following SARS-CoV-2 infection [2,5].

This distinction is important, as it indicates that SARS-CoV-2 infection may be associated with metabolic dysregulation in susceptible individuals.

Overall, our results are consistent with the involvement of β-cell dysfunction in both acute and post-COVID metabolic disturbance and suggest that post-COVID metabolic alterations may differ from classical MetS, particularly in individuals presenting with newly developed dysglycaemia after infection. However, these observations should be interpreted with caution given the cross-sectional design of the study.

### 4.3. Thyroid Hormone Alterations in COVID-19

In addition to metabolic differences, the present study identified significant alterations in thyroid hormone parameters across the investigated groups. Patients with active COVID-19 exhibited markedly lower FT3 concentrations than both the post-COVID cohort and the MetS reference population. These findings are consistent with previous reports describing altered thyroid hormone metabolism during acute SARS-CoV-2 infection [11,12,13].

Several studies have reported abnormalities in thyroid function among hospitalized patients with COVID-19, including reduced circulating T3 levels, suppressed TSH concentrations, and transient thyrotoxicosis [11,12,13]. In many cases, these hormonal changes resemble NTIS, characterized by reduced peripheral conversion of T4 to T3 during severe systemic illness [10]. Accordingly, the reduced FT3 levels observed in the active COVID-19 group in the present study are likely to reflect systemic metabolic adaptation rather than primary thyroid gland pathology. Differences in thyroid status across groups should also be interpreted with caution, as acute illness-related changes, such as euthyroid sick syndrome observed in patients with active COVID-19, are not directly comparable with chronic thyroid dysfunction.

Consistent with this interpretation, the FT3/FT4 ratio was also significantly lower during acute COVID-19 infection. Because this ratio is commonly considered an indirect marker of peripheral T4-to-T3 conversion, its reduction further supports the presence of impaired peripheral thyroid hormone metabolism during acute illness [10].

The mechanisms underlying these changes are likely multifactorial. Systemic inflammation may suppress deiodinase activity and reduce peripheral conversion of T4 to T3, while hypothalamic-pituitary-thyroid axis dysregulation and possible direct thyroid involvement may also contribute [10,25].

Importantly, FT3 concentrations in the post-COVID cohort were substantially higher than those observed during active infection and were comparable to those in individuals with MetS. By contrast, FT4 concentrations were significantly lower in the post-COVID cohort than in both the active COVID-19 and MetS groups. Although FT3 levels appeared largely restored after recovery from infection, the lower FT4 may indicate possible alterations in thyroid hormone regulation or turnover during the post-infectious phase. At present, it remains unclear whether this represents transient endocrine adaptation or a more persistent feature of post-COVID metabolic disturbance [12,16].

Taken together, these observations are consistent with the involvement of thyroid hormone dynamics as a component of the systemic endocrine response to SARS-CoV-2 infection. Reduced FT3 availability during acute illness likely reflects adaptive metabolic regulation, whereas the heterogeneity of thyroid findings in the post-COVID state suggests more complex interactions among thyroid regulation, metabolic homeostasis, and recovery-related changes in endocrine regulation.

### 4.4. Thyroid Dysfunction in the Post-COVID Cohort

In the present study, thyroid dysfunction was observed in a notable proportion of individuals evaluated after recovery from COVID-19. Importantly, however, the defining feature of this group was newly identified disturbance in carbohydrate metabolism following SARS-CoV-2 infection, whereas thyroid assessment was performed as part of a broader endocrine evaluation. Therefore, the observed prevalence of thyroid abnormalities reflects findings in a metabolically characterized post-COVID population rather than a cohort selected on the basis of thyroid disease.

Importantly, when thyroid status was evaluated based solely on biochemical thyroid function categories, excluding clinical history and euthyroid sick syndrome, no significant differences in the prevalence of thyroid dysfunction were observed across groups. This finding suggests that the apparent between-group differences in thyroid dysfunction may be influenced by the inclusion of pre-existing thyroid disease and the high prevalence of euthyroid sick syndrome during acute COVID-19, rather than reflecting true differences in intrinsic thyroid pathology.

Within the post-COVID group, subgroup analyses demonstrated heterogeneity in thyroid status. Significant differences were observed in TSH concentrations and thyroid autoimmunity markers across individuals without thyroid dysfunction, those with pre-existing thyroid disease, and those with newly detected thyroid abnormalities. In particular, anti-thyroid peroxidase (Anti-TPO) antibody concentrations were markedly elevated in participants with newly detected thyroid dysfunction as well as in those with pre-existing thyroid disease, suggesting a possible autoimmune component in a subset of post-COVID patients. Alternative TSH thresholds (e.g., >2.5 mIU/L) have been proposed in relation to thyroid autoimmunity and metabolic risk; however, standard laboratory reference ranges were used in the present study to ensure comparability with routine clinical practice.

Previous studies have reported thyroid dysfunction during and after COVID-19 infection, including transient thyrotoxicosis, thyroiditis, and autoimmune thyroid disease [11,12,26]. Viral infections are well-recognized triggers of autoimmune thyroid disorders through mechanisms such as molecular mimicry, immune activation, and bystander inflammation [25]. Several reports have also described subacute thyroiditis or newly diagnosed autoimmune thyroid disease following SARS-CoV-2 infection, suggesting that SARS-CoV-2 infection may be associated with the onset of thyroid dysfunction in susceptible individuals [27,28].

At the same time, crude subgroup comparisons did not reveal significant differences in FT3, FT4, the FT3/FT4 ratio, or β-cell compensation between thyroid-status subgroups. After adjustment for demographic and metabolic covariates, modest differences in FT4 became apparent, suggesting subtle endocrine heterogeneity; however, these findings should be interpreted cautiously. Although the highest proportion of β-cell dysfunction was observed in individuals with newly detected thyroid abnormalities, this difference was not statistically significant.

Overall, these findings are consistent with thyroid dysfunction representing a heterogeneous endocrine feature among individuals with post-COVID metabolic disturbance and may reflect broader endocrine alterations associated with SARS-CoV-2 infection in a metabolically vulnerable population.

### 4.5. Thyroid Hormones and β-Cell Function

An important observation of the present study was the association between peripheral thyroid hormone activity and β-cell compensatory capacity within the post-COVID cohort. Specifically, circulating FT3 concentrations and the FT3/FT4 ratio were positively correlated with HOMA-B/HOMA-IR, with higher FT3 availability being associated with greater β-cell compensation relative to IR, whereas TSH concentrations and thyroid autoantibodies were not significantly associated with indices of β-cell function. These findings suggest that peripheral thyroid hormone activity, rather than TSH alone, may better reflect metabolic regulation in the post-COVID state.

These findings are biologically plausible given the established role of thyroid hormones in metabolic regulation. T3, the biologically active thyroid hormone, influences hepatic glucose production, peripheral insulin sensitivity, and pancreatic β-cell activity [6,7]. Experimental studies have also shown that T3 modulates insulin secretion and mitochondrial energy metabolism within pancreatic β-cells, thereby affecting their ability to respond to metabolic demand [8,9]. In this context, the observed association between FT3-related indices and HOMA-B/HOMA-IR is consistent with the established role of thyroid hormones in glucose metabolism and metabolic adaptation.

The association between FT3 and β-cell compensation observed in the present study may also reflect the importance of peripheral thyroid hormone conversion. The FT3/FT4 ratio is commonly considered an indirect indicator of deiodinase activity and tissue availability of active thyroid hormone. Reduced conversion of T4 to T3 has been described in several metabolic and inflammatory conditions and may be associated with impaired metabolic regulation [10,29]. In the context of COVID-19, alterations in deiodinase activity and thyroid hormone metabolism during illness and recovery may therefore influence metabolic homeostasis [10,12].

Although the cross-sectional design does not allow for causal inference, the observed associations suggest that thyroid hormone dynamics may be linked to β-cell functional reserve in the post-COVID metabolic state. Reduced FT3 availability could be related to differences in β-cell compensatory capacity, potentially through effects on mitochondrial energy metabolism and insulin secretory responses, whereas preserved peripheral T3 generation may support more effective metabolic adaptation [8,9].

Taken together, these findings are consistent with a potential role of thyroid hormone activity—particularly circulating FT3 levels and indices of peripheral conversion—as a component of a broader pattern of post-COVID immunometabolic alterations that may reflect a distinct, yet not fully characterized, phenotype.

### 4.6. Cytokine Findings and Immunometabolic Context

Because COVID-19 is characterized by systemic immune activation, we further explored whether post-COVID thyroid phenotypes were associated with differences in circulating cytokine profiles. In the present study, concentrations of TNF-α, IFN-γ, IL-17A, IL-10, and IL-7 did not differ significantly across thyroid status subgroups within the post-COVID cohort. These findings indicate that the heterogeneity in thyroid function observed among post-COVID participants was not clearly accompanied by detectable differences in systemic cytokine concentrations.

Previous studies have demonstrated that acute COVID-19 may be associated with elevated inflammatory mediators, including TNF-α, IL-6, and other cytokines involved in systemic immune activation [30,31]. Cytokine signaling has also been implicated in the regulation of thyroid hormone metabolism and hypothalamic–pituitary–thyroid axis activity, particularly during critical illness [10]. Pro-inflammatory cytokines may suppress deiodinase activity and reduce peripheral conversion of T4 to T3, contributing to the low-T3 pattern frequently observed in severe systemic disease.

However, the absence of significant cytokine differences between thyroid status suggests that systemic cytokine concentrations measured in the present study may not fully account for the thyroid alterations observed in the post-COVID metabolic state. This observation is consistent with emerging evidence indicating that post-acute sequelae of COVID-19 may involve persistent but heterogeneous immune activation that is not always reflected by conventional circulating cytokine profiles [32]. Instead, these endocrine changes may be related to a combination of metabolic adaptation, immune regulation, and recovery-related changes in endocrine function following SARS-CoV-2 infection.

Importantly, the cytokine analysis performed in this study was exploratory and involved relatively small subgroup sizes. Therefore, these findings should be interpreted cautiously and considered primarily hypothesis-generating. Future studies integrating comprehensive immune profiling with endocrine and metabolic phenotyping will be necessary to clarify the potential contribution of inflammatory pathways to post-COVID thyroid and metabolic dysfunction.

### 4.7. Clinical Implications

The findings of this study have several potential clinical implications. First, they indicate that post-COVID metabolic disturbances may be accompanied by alterations in thyroid hormone parameters that extend beyond classical thyroid disease categories. The observed associations between FT3-related indices and β-cell compensatory capacity raise the possibility that thyroid hormone activity may be linked to metabolic adaptation following SARS-CoV-2 infection.

Second, the observed prevalence of thyroid abnormalities in the post-COVID cohort suggests that thyroid assessment may be of value in individuals presenting with newly identified metabolic disturbances after COVID-19. Although these abnormalities may not always reflect overt thyroid disease, thyroid hormone alterations and thyroid autoimmunity may represent additional features of post-COVID endocrine changes.

Finally, the results are consistent with the possibility that post-COVID metabolic patterns may differ from classical MetS. Clinical evaluation of individuals with post-COVID metabolic disturbance may therefore benefit from a broader endocrine assessment, including both metabolic and thyroid-related parameters.

These observations highlight the potential relevance of integrating thyroid hormone assessment into the clinical evaluation of post-COVID metabolic disturbances; however, further studies are required to establish clinical utility.

### 4.8. Strengths and Limitations

The present study has several notable strengths. First, it provides a comprehensive characterization of metabolic and thyroid parameters across three clinically distinct states—active COVID-19 infection, post-COVID metabolic disturbances, and COVID-negative MetS—allowing contextual interpretation of endocrine alterations in relation to both acute infection and established metabolic disease.

Second, the study adopts an integrative immunometabolic approach by simultaneously evaluating β-cell compensatory capacity, thyroid hormone dynamics, and thyroid autoimmunity within the same cohort. This combined assessment offers novel insight into the potential interplay between pancreatic and thyroid function in the context of COVID-19-related metabolic disturbance.

Third, the inclusion of a post-COVID cohort defined by newly developed disturbances in carbohydrate metabolism following SARS-CoV-2 infection represents a clinically relevant and well-characterized population, enabling focused investigation of post-infectious metabolic alterations. In addition, the use of both biochemical and clinical criteria for thyroid assessment reflects real-world endocrine practice and enhances the clinical relevance of the findings.

Despite these strengths, several limitations should be acknowledged. First, the cross-sectional design precludes causal inference regarding the observed associations between thyroid hormone dynamics and β-cell compensatory capacity. The findings should therefore be interpreted as descriptive and hypothesis-generating rather than indicative of causal relationships.

Second, the study groups were not matched for key demographic and clinical characteristics, most notably age. Participants in the active COVID-19 group were older than those in the post-COVID and MetS groups, which may have influenced metabolic and endocrine parameters. Age differences between groups, particularly the older age of the active COVID-19 cohort, may have influenced β-cell function and thyroid parameters despite statistical adjustment.

Third, the grouping strategy was based on clinically distinct metabolic contexts rather than matched populations. In particular, the post-COVID cohort was defined by newly developed disturbances in carbohydrate metabolism following SARS-CoV-2 infection, resulting in inherent heterogeneity and limiting direct comparability with the other groups. Consequently, between-group comparisons should be interpreted within the context of this exploratory design.

Fourth, the definition of thyroid dysfunction was based on a composite clinical approach incorporating biochemical parameters, thyroid autoimmunity, and clinical history. While this approach reflects real-world clinical assessment, it introduces complexity in interpretation, particularly when comparing across different clinical states. In addition, the inclusion of euthyroid sick syndrome in the active COVID-19 group further limits direct comparability with the other groups.

Fifth, the sample size was relatively modest, particularly for subgroup and cytokine analyses, which may have limited statistical power to detect subtle differences and increased the risk of type II error.

Sixth, cytokine measurements were available only in a subset of participants and were included as an exploratory component of the study. These findings should therefore be interpreted with caution and require confirmation in larger, adequately powered studies.

Finally, the study population consisted of unvaccinated individuals, which may limit the generalizability of the findings to current populations with widespread COVID-19 vaccination and evolving viral variants.

Taken together, these limitations suggest that the present findings should be interpreted cautiously and primarily as exploratory observations describing potential immunometabolic interactions in post-COVID metabolic disturbances.

## 5. Conclusions

In conclusion, this study demonstrates that post-COVID metabolic disturbances are characterized by impaired β-cell compensatory capacity and altered thyroid hormone dynamics. Individuals with active COVID-19 exhibited the lowest β-cell compensation and reduced FT3-related indices, whereas patients evaluated after recovery from infection demonstrated an intermediate metabolic profile relative to the MetS reference group.

Within the post-COVID cohort, circulating FT3 concentrations and the FT3/FT4 ratio were positively associated with β-cell compensatory capacity, suggesting that peripheral thyroid hormone activity may contribute to metabolic adaptation following SARS-CoV-2 infection. Although thyroid abnormalities and thyroid autoimmunity were relatively common in the post-COVID population, thyroid antibody levels were not associated with β-cell function.

Overall, these findings support the hypothesis that post-COVID metabolic disturbance may represent a distinct immunometabolic phenotype characterized by altered β-cell compensation and thyroid hormone metabolism. Further longitudinal studies are needed to clarify the mechanisms linking thyroid hormone dynamics with pancreatic β-cell function and to determine whether thyroid-related pathways may contribute to metabolic risk stratification after COVID-19.

## Figures and Tables

**Table 1:** Baseline demographic and clinical characteristics of the study population.

Variable	Group 1 Active COVID (n = 32)	Group 2 Post-COVID (n = 35)	Group 3 COVID (−) MetS (n = 33)	Test Statistic	*p*-Value ^†^
Age (years), mean ± SD (range)	66.5 ± 13.1 (46–87)	45.4 ± 14.3 (22–71)	48.2 ± 12.7 (21–68)	H(2) = 30.28	<0.001
Sex, n (%)				χ^2^(2) = 16.38	<0.001
Male	18 (56.3%)	12 (34.3%)	3 (9.1%)		
Female	14 (43.7%)	23 (65.7%)	30 (90.9%)		
BMI (kg/m^2^), mean ± SD	NA	31.9 ± 7.4	35.7 ± 7.9	U = 407.0	0.054
Waist circumference (cm), mean ± SD	NA	104.8 ± 15.8	108.8 ± 17.7	U = 459.5	0.205
Hypertension, n (%)	13 (40.6%)	11 (31.4%)	16 (48.5%)	χ^2^(2) = 2.96	0.228
Dyslipidemia, n (%)	10 (31.3%)	14 (40.0%)	12 (36.4%)	χ^2^(2) = 0.55	0.76
Antihypertensive therapy, n (%)	6 (18.8%)	7 (20.0%)	16 (48.5%)	χ^2^(2) = 6.42	0.040
Statin therapy, n (%)	7 (21.9%)	0 (0.0%)	7 (21.2%)	χ^2^(2) = 8.87	0.012
Current smokers, n (%)	7 (21.9%)	8 (22.9%)	10 (30.3%)	χ^2^(2) = 0.75	0.688
Family history of diabetes mellitus, n (%)	7 (21.9%)	14 (40.0%)	15 (45.5%)	χ^2^(2) = 3.82	0.148
Family history of thyroid disease, n (%)	9 (28.1%)	7 (20.0%)	11 (33.3%)	χ^2^(2) = 1.48	0.477

Data are presented as mean ± standard deviation (SD) for continuous variables and number (percentage) for categorical variables. ^†^ *p*-values correspond to the test statistics presented in the adjacent column. Continuous variables were compared using the Kruskal–Wallis H test or Mann–Whitney U test, as appropriate, and categorical variables were compared using the chi-square (χ^2^) test. BMI and waist circumference were not available for patients with active COVID-19 due to clinical conditions during acute hospitalization. NA—not available. Dyslipidemia was defined based on abnormal lipid profile results from laboratory testing, documented diagnosis in medical records, and/or current use of lipid-lowering therapy, including statins. Hypertension was defined based on documented diagnosis in medical records and/or current use of antihypertensive medication. Current smokers were defined as individuals reporting active tobacco use at the time of study enrolment.

**Table 2:** Distribution of glucose metabolism disorders and antidiabetic therapy across the study groups.

Variable	Group 1 Active COVID (n = 32)	Group 2 Post-COVID (n = 35)	Group 3 COVID (−) MetS (n = 33)
Diabetes mellitus, n (%)	16 (50.0%)	19 (54.3%)	11 (33.3%)
Newly diagnosed diabetes, n (%)	7 (21.9%)	19 (54.3%)	2 (6.1%)
Type 1 diabetes/LADA, n (%)	—	8 (22.9%)	—
Type 2 diabetes, n (%)	9 (28.1%)	11 (31.4%)	11 (33.3%)
Prediabetes, n (%)	—	7 (20.0%)	7 (21.2%)
IR with normoglycaemia, n (%)	—	9 (25.7%)	15 (45.5%)
Insulin therapy, n (%)	1 (3.1%)	—	4 (12.1%)
Oral antidiabetic therapy, n (%)	8 (25.0%)	—	5 (15.2%)

Data are presented as number (percentage). Diabetes mellitus and prediabetes were diagnosed according to the World Health Organization (WHO) criteria. Prediabetes included impaired fasting glucose (IFG) and impaired glucose tolerance (IGT). Insulin resistance (IR) with normoglycaemia was defined based on elevated fasting and/or stimulated insulin levels in the presence of normal glucose values. Information on antidiabetic therapy was obtained from medical records at the time of hospitalization or study evaluation. LADA—latent autoimmune diabetes in adults. MetS—Metabolic syndrome.

**Table 3:** Baseline metabolic and thyroid function characteristics of the study population.

Variable Mean ± SD Median (Q1–Q3)	Group 1 Active COVID (n = 32)	Group 2 Post-COVID (n = 35)	Group 3 COVID (−) MetS (n = 33)	Test Statistic	*p*-Value ^†^
β-cell dysfunction, n (%)	27 (83.9%)	21 (59.4%)	8 (24.0%)	χ^2^(2) = 20.40	<0.001
HOMA-B/HOMA-IR	21.80 ± 15.53 16.89 (11.90–23.19)	45.66 ± 51.20 27.27 (13.65–56.33)	74.33 ± 53.80 65.60 (34.78–92.03)	H(2) = 22.17	<0.001
TSH [mIU/L]	1.40 ± 1.22 1.03 (0.49–2.14)	2.76 ± 1.62 2.40 (1.80–2.99)	4.93 ± 11.19 2.50 (1.74–3.02)	H(2) = 20.59	<0.001
FT3 [pmol/L]	3.02 ± 0.93 2.81 (2.29–3.54)	4.67 ± 0.83 4.58 (4.00–5.40)	4.90 ± 0.85 4.80 (4.17–5.41)	H(2) = 47.03	<0.001
FT4 [pmol/L]	17.78 ± 4.12 17.87 (14.91–20.71)	14.61 ± 2.79 14.20 (12.48–16.80)	16.84 ± 2.83 17.07 (15.01–18.89)	H(2) = 14.83	<0.001
TPOAb [IU/mL]	36.69 ± 29.23 21.50 (17.00–51.25)	296.32 ± 737.25 48.00 (20.50–119.25)	120.67 ± 261.61 47.00 (17.00–69.00)	H(2) = 3.33	0.190
TgAb (TAT) [IU/mL]	41.78 ± 36.90 27.50 (19.00–45.25)	73.23 ± 90.14 37.75 (14.46–95.21)	60.36 ± 58.06 45.00 (18.00–70.00)	H(2) = 0.54	0.765
FT3/FT4 Ratio	0.18 ± 0.09 0.17 (0.12–0.21)	0.33 ± 0.08 0.32 (0.26–0.38)	0.30 ± 0.08 0.29 (0.26–0.31)	H(2) = 42.94	<0.001
Thyroid dysfunction, n (%)	7 (21.9%)	20 (57.1%)	17 (53.1%)	χ^2^(2) = 9.86	0.007
Newly diagnosed thyroid dysfunction, n (%)	3 (9.4%)	6 (17.6%)	6 (18.8%)	χ^2^(2) = 3.12	0.21
LT4 therapy, n (%)	4 (12.5%)	3 (8.8%)	4 (12.5%)	χ^2^(2) = 0.61	0.74

Values are presented as mean ± SD and median (Q1–Q3). Continuous variables were compared using the Kruskal–Wallis test. Categorical variables were compared using the χ^2^ test. β-cell dysfunction was defined as HOMA-B/HOMA-IR < 34.8, corresponding to the 25th percentile of the COVID-negative MetS reference group (G3). ^†^ Statistical significance was defined as *p* < 0.05.

**Table 4:** Adjusted GLM analysis for thyroid parameters across study groups.

Variable	Comparison	Β	Exp(β)	95% CI	*p*-Value ^†^
TSH	G1 vs. G3	−1.08	0.34	0.15–0.78	0.011
	G2 vs. G3	−0.57	0.57	0.28–1.13	0.106
	G1 vs. G2	−0.51	0.60	0.27–1.35	0.218
FT3	G1 vs. G3	−0.44	0.65	0.56–0.74	<0.001
	G2 vs. G3	−0.05	0.95	0.85–1.07	0.388
	G1 vs. G2	−0.39	0.68	0.60–0.78	<0.001
FT4	G1 vs. G3	0.08	1.08	0.96–1.22	0.194
	G2 vs. G3	−0.14	0.87	0.79–0.96	0.007
	G1 vs. G2	0.22	1.24	1.10–1.40	<0.001
FT3/FT4 ratio	G1 vs. G3	−0.43	0.65	0.53–0.80	<0.001
	G2 vs. G3	0.10	1.10	0.93–1.31	0.249
	G1 vs. G2	−0.53	0.59	0.49–0.72	<0.001
TgAb (TAT)	G1 vs. G3	0.00	1.00	0.56–1.78	0.998
	G2 vs. G3	0.26	1.30	0.80–2.09	0.286
	G1 vs. G2	−0.26	0.77	0.44–1.36	0.370
TPOAb	G1 vs. G3	−0.47	0.62	0.20–1.90	0.406
	G2 vs. G3	0.81	2.26	0.90–5.67	0.083
	G1 vs. G2	−1.29	0.28	0.09–0.82	0.021

Data are derived from generalised linear models (GLM) with Gamma distribution and log link, adjusted for age and sex. β represents the regression coefficient on the log scale and Exp(β) the exponentiated coefficient (ratio of means). MetS (G3) was used as the reference group where applicable. Values are presented with 95% confidence intervals (CI). ^†^ Statistical significance was defined as *p* < 0.05.

**Table 5:** Thyroid function * categories across study groups.

Thyroid Status	Group 1 Active COVID (n = 32)	Group 2 Post-COVID (n = 35)	Group 3 COVID (−) MetS (n = 33)
Euthyroidism	15 (46.9%)	29 (82.9%)	29 (87.9%)
Subclinical hypothyroidism	0 (0.0%)	3 (8.6%)	3 (9.1%)
Overt hypothyroidism	1 (3.1%)	2 (5.7%)	1 (3.0%)
Subclinical hyperthyroidism	1 (3.1%)	0 (0.0%)	0 (0.0%)
Overt hyperthyroidism	1 (3.1%)	1 (2.9%)	0 (0.0%)
Euthyroid sick syndrome (low T3 syndrome)	14 (43.8%)	0 (0.0%)	0 (0.0%)

* Thyroid function status was classified according to biochemical criteria using the laboratory reference ranges for TSH (0.20–4.20 mIU/L), FT3 (3.10–6.80 pmol/L), and FT4 (11.9–21.6 pmol/L). Euthyroidism was defined as normal TSH, FT3, and FT4 concentrations. Subclinical hypothyroidism was defined as elevated TSH with normal FT4, while overt hypothyroidism was defined as elevated TSH with reduced FT4. Subclinical hyperthyroidism was defined as suppressed TSH with normal FT3 and FT4, whereas overt hyperthyroidism was defined as suppressed TSH with elevated FT3 and/or FT4. Euthyroid sick syndrome was defined as reduced FT3 with normal TSH and FT4 levels.

**Table 6:** Distribution of thyroid status within Post-COVID cohort (Group 2) subgroups.

Thyroid Status	No TD (n = 14)	PTD (n = 9)	NDTD (n = 12)
Euthyroidism	14 (100%)	5 (55.6%)	10 (83.3%)
Subclinical hypothyroidism	0 (0.0%)	2 (22.2%)	1 (8.3%)
Overt hypothyroidism	0 (0.0%)	1 (11.1%)	1 (8.3%)
Overt hyperthyroidism	0 (0.0%)	1 (11.1%)	0 (0.0%)
Euthyroid sick syndrome (low T3)	0 (0.0%)	0 (0.0%)	0 (0.0%)

TD = thyroid dysfunction; PTD = pre-existing thyroid disease; NDTD = newly detected thyroid dysfunction.

**Table 7:** Comparison of β-cell function, thyroid function, and thyroid autoimmunity markers among thyroid status subgroups within the post-COVID cohort.

Variable Mean ± SD Median (Q1–Q3)	No TD (n = 14)	PTD (n = 9)	NDTD (n = 12)	Test Statistic ^†^
β-cell dysfunction, n (%)	7 (50.0%)	4 (44.4%)	10 (83.3%)	χ^2^(2) = 4.21 *p* = 0.122
HOMA-B/HOMA-IR	35.28 ± 30.36	56.81 ± 59.91	39.67 ± 60.83	H(2) = 1.80 *p* = 0.406
	28.22 (11.32–56.95)	35.89 (16.02–66.39)	23.08 (13.41–24.61)	
TSH [mIU/L]	2.41 ± 0.65	4.01 ± 1.48	2.23 ± 2.08	H(2) = 11.53 *p* = 0.003
	2.35 (1.96–2.91)	4.10 (2.80–4.80)	1.77 (1.55–2.13)	
FT3 [pmol/L]	4.74 ± 0.77	4.50 ± 0.94	4.72 ± 0.87	H(2) = 0.68 *p* = 0.711
	4.76 (4.09–5.35)	4.50 (3.79–4.90)	4.54 (4.08–5.39)	
FT4 [pmol/L]	13.94 ± 2.39	13.84 ± 2.93	15.99 ± 2.82	H(2) = 4.35 *p* = 0.113
	13.44 (12.45–14.56)	13.30 (12.10–16.80)	16.57 (14.05–17.45)	
FT3/FT4 ratio	0.34 ± 0.09	0.33 ± 0.10	0.29 ± 0.07	H(2) = 3.46 *p* = 0.177
	0.33 (0.29–0.38)	0.31 (0.27–0.38)	0.28 (0.24–0.33)	
TgAb (TAT) [IU/mL]	28.10 ± 25.18	117.67 ± 134.17	88.79 ± 79.39	H(2) = 6.19 *p* = 0.045
	19.86 (13.00–41.00)	63.00 (22.00–167.00)	67.50 (29.52–106.25)	
TPOAb [IU/mL]	19.41 ± 21.03	514.11 ± 987.48	758.67 ± 1105.89	H(2) = 12.27 *p* = 0.002
	13.60 (2.00–33.00)	96.50 (57.50–316.15)	227.00 (123.50–1129.00)	

Values are presented as mean ± SD and median (Q1–Q3). Continuous variables were compared using the Kruskal–Wallis test. Categorical variables were compared using the χ^2^ test. ^†^ Statistical significance was defined as *p* < 0.05. TD = thyroid dysfunction; PTD = pre-existing thyroid disease; NDTD = newly detected thyroid dysfunction.

**Table 8:** Cytokine profile across thyroid subgroups in the post-COVID cohort (Group 2).

Variable Mean ± SD Median (Q1–Q3)	No TD (n = 14 ^†^)	PTD (n = 9 ^†^)	NDTD (n = 12 ^†^)	Test Statistic
IL-7 [pg/mL]	41.27 ± 52.28 27.45 (22.30–33.22)	24.64 ± 12.81 22.69 (15.64–33.28)	31.08 ± 16.59 27.50 (22.31–33.40)	H(2) = 0.43 *p* = 0.808
	n = 13	n = 8	n = 7	
IL-10 [pg/mL]	3.69 ± 4.26 2.48 (1.93–3.50)	2.77 ± 1.61 2.34 (1.88–3.02)	3.21 ± 2.09 2.75 (2.04–3.62)	H(2) = 0.44 *p* = 0.802
	n = 14	n = 8	n = 7	
IL-17A [pg/mL]	35.11 ± 22.01 27.11 (25.17–29.04)	25.49 ± 12.12 25.08 (18.84–28.44)	32.41 ± 17.30 26.91 (24.77–34.65)	H(2) = 2.15 *p* = 0.342
	n = 13	n = 8	n = 7	
IFN-γ [pg/mL]	5.78 ± 4.58 4.07 (3.07–6.63)	4.48 ± 2.54 3.91 (2.99–5.46)	6.18 ± 3.70 4.78 (3.92–7.31)	H(2) = 2.00 *p* = 0.368
	n = 14	n = 8	n = 7	
TNF-α [pg/mL]	98.11 ± 76.24 84.31 (46.11–105.77)	69.54 ± 36.92 61.92 (43.16–91.43)	103.89 ± 72.31 86.27 (55.22–118.04)	H(2) = 2.19 *p* = 0.334
	n = 14	n = 8	n = 7	

Values are presented as mean ± SD and median (Q1–Q3). *p*-values were calculated using the Kruskal–Wallis test. ^†^ Sample size (n) varies across cytokines due to sample availability and is indicated for each variable. Statistical significance was defined as *p* < 0.05. TD = thyroid dysfunction; PTD = pre-existing thyroid disease; NDTD = newly detected thyroid dysfunction.

## Data Availability

Data are available from the corresponding author on reasonable request.

## Supplementary Materials

The following supporting information can be downloaded at https://www.mdpi.com/article/10.3390/biomedicines14071420/s1, Table S1: Dunn post hoc pairwise comparisons for HOMA-B/HOMA-IR across study groups; Table S2. Adjusted GLM analysis for β-cell compensation (HOMA-B/HOMA-IR); Table S3. Dunn post hoc pairwise comparisons for metabolic and thyroid parameters across study groups; Table S4. Dunn post hoc pairwise comparisons within the post-COVID thyroid subgroups; Table S5. Adjusted GLM analysis across thyroid status subgroups within the post-COVID cohort; Table S6. Spearman correlations between thyroid markers and β-cell function in the post-COVID cohort.

## Abbreviations

The following abbreviations are used in this manuscript:

BMI	Body mass index
CI	Confidence interval
DM	Diabetes mellitus
ECLIA	Electrochemiluminescence immunoassay
ELISA	Enzyme-linked immunosorbent assay
FT3	Free triiodothyronine
FT4	Free thyroxine
GLM	Generalized linear model
HbA1c	Glycated haemoglobin
HOMA-B	Homeostasis model assessment of β-cell function
HOMA-IR	Homeostasis model assessment of insulin resistance
IFG	Impaired fasting glucose
IFN-γ	Interferon-γ
IGT	Impaired glucose tolerance
IL-10	Interleukin-10
IL-17A	Interleukin-17A
IL-7	Interleukin-7
IQR	Interquartile range
IR	Insulin resistance
LADA	Latent autoimmune diabetes in adults
MetS	Metabolic syndrome
NTIS	Non-thyroidal illness syndrome (euthyroid sick syndrome, low T3 syndrome)
OGTT	Oral glucose tolerance test
PCR	Polymerase chain reaction
T1DM	Type 1 diabetes mellitus
T2DM	Type 2 diabetes mellitus
TgAb (TAT)	Thyroglobulin antibodies
TNF-α	Tumor necrosis factor-α
TPOAb	Thyroid peroxidase antibodies
TSH	Thyroid-stimulating hormone
