# Supplementary material for: β-Cell Dysfunction and Altered Thyroid Hormone Dynamics in Post-COVID Metabolic Disturbances: An Immunometabolic Cross-Sectional Study
Source: Biomedicines. 2026 Jun 23;14(7):1420. doi: 10.3390/biomedicines14071420 (PMC13403416; doi:10.3390/biomedicines14071420)
Supplement: Supplementary file 1 [file biomedicines-14-01420-s001.zip › biomedicines-4282007-supplementary.pdf]

biomedicines

MDPI

Supplementary Tables

Supplementary Table S1. Dunn post hoc pairwise comparisons for HOMA-B/HOMA-IR across study groups

Variable	Comparison	Z statistic	Adjusted *p*-value†
**HOMA-B /HOMA-IR**	G1 Active COVID vs G2 Post-COVID	-3.42	**<0.001**
	G1 Active COVID vs G3 Metabolic syndrome	-4.71	**<0.001**
	G2 Post-COVID vs G3 Metabolic syndrome	-2.57	**0.010**

* Statistical test: Dunn post hoc test with Bonferroni correction following Kruskal–Wallis analysis.
† Statistical significance was defined as $p < 0.05$.

Supplementary Table S2. Adjusted GLM analysis for β-cell compensation (HOMA-B/HOMA-IR)

Variable	Comparison	β	Exp(β)	95% CI	*p*-value†
**HOMA-B/ HOMA-IR**	G1 vs G3	−1.238	0.29	0.19 – 0.44	**<0.001**
	G2 vs G3	−0.478	0.62	0.43 – 0.88	**0.009**
	G1 vs G2	−0.755	0.47	0.32 – 0.70	**<0.001**

*** Data are derived from generalised linear models (GLM) with Gamma distribution and log link, adjusted for age and sex**. β represents the regression coefficient on the log scale and Exp(β) the exponentiated coefficient (ratio of means). Metabolic Syndrome (G3) was used as the reference group where applicable. Values are presented with 95% confidence intervals (CI).
† Statistical significance was defined as $p < 0.05$.

Supplementary Table S3. Dunn post hoc pairwise comparisons for metabolic and thyroid parameters across study groups

Variable	Comparison	Z statistic	Adjusted *p*-value†
**TSH**	G1 Active COVID vs G2 Post-COVID	-3.94	**<0.001**
	G1 Active COVID vs G3 Metabolic syndrome	-3.96	**<0.001**
	G2 Post-COVID vs G3 Metabolic syndrome	-0.08	0.936
**FT3**	G1 Active COVID vs G2 Post-COVID	-5.51	**<0.001**
	G1 Active COVID vs G3 Metabolic syndrome	-6.34	**<0.001**
	G2 Post-COVID vs G3 Metabolic syndrome	-0.93	0.354
**FT4**	G1 Active COVID vs G2 Post-COVID	3.55	**<0.001**
	G1 Active COVID vs G3 Metabolic syndrome	0.55	0.585
	G2 Post-COVID vs G3 Metabolic syndrome	-3.02	**0.003**

**FT3/FT4 ratio**	G1 Active COVID vs G2 Post-COVID	-6.22	**<0.001**
	G1 Active COVID vs G3 Metabolic syndrome	-4.97	**<0.001**
	G2 Post-COVID vs G3 Metabolic syndrome	1.19	0.233
**TgAb (TAT)**	G1 Active COVID vs G2 Post-COVID	-0.39	0.696
	G1 Active COVID vs G3 Metabolic syndrome	-0.73	0.464
	G2 Post-COVID vs G3 Metabolic syndrome	-0.35	0.726
**TPOAb**	G1 Active COVID vs G2 Post-COVID	-1.79	0.074
	G1 Active COVID vs G3 Metabolic syndrome	-1.23	0.220
	G2 Post-COVID vs G3 Metabolic syndrome	0.56	0.579

* Statistical test: Dunn post hoc test with Bonferroni correction following Kruskal–Wallis analysis.
† Statistical significance was defined as $p < 0.05$.

Supplementary Table S4. Dunn post hoc pairwise comparisons within the post-COVID thyroid subgroups

**Variable**	**Comparison**	**Z statistic**	**Adjusted *p*-value**†
**TSH**	PTD vs NDTD	3.39	**0.002**
	PTD vs No TD	2.17	0.089
	NDTD vs No TD	−1.44	0.454
**TPOAb**	PTD vs NDTD	0.10	1.000
	PTD vs No TD	3.07	**0.006**
	NDTD vs No TD	3.22	**0.004**
**TgAb (TAT)**	PTD vs NDTD	−0.18	1.000
	PTD vs No TD	1.92	0.166
	NDTD vs No TD	2.27	0.070

* Statistical test: Pairwise comparisons were performed using Dunn's post hoc test with Bonferroni correction following significant Kruskal–Wallis tests.
† Statistical significance was defined as $p < 0.05$.
No TD = no thyroid dysfunction; PTD = pre-existing thyroid disease; NDTD = newly detected thyroid dysfunction

Supplementary Table S5. Adjusted GLM analysis across thyroid status subgroups within the post-COVID cohort

Variable	Comparison	Β	Exp(β)	95% CI	*p*-value†
**HOMA-B/HOMA-IR**	PTD vs No TD	0.06	1.06	0.35 – 3.24	0.914
	NDTD vs No TD	−0.50	0.61	0.24 – 1.56	0.301
	PTD vs NDTD	0.56	1.75	0.66 – 4.61	0.258
**TSH**	PTD vs No TD	0.29	1.33	0.77 – 2.32	0.310
	NDTD vs No TD	−0.32	0.72	0.45 – 1.16	0.182
	PTD vs NDTD	0.61	1.84	1.14 – 2.98	**0.013**
**FT3**	PTD vs No TD	−0.04	0.96	0.80 – 1.15	0.638
	NDTD vs No TD	0.01	1.01	0.86 – 1.18	0.906
	PTD vs NDTD	−0.05	0.95	0.81 – 1.11	0.511
**FT4**	PTD vs No TD	−0.08	0.92	0.76 – 1.12	0.423
	NDTD vs No TD	0.10	1.11	0.94 – 1.30	0.230
	PTD vs NDTD	−0.18	0.84	0.71 – 0.99	**0.035**
**FT3/FT4 ratio**	PTD vs No TD	0.05	1.06	0.81 – 1.37	0.684
	NDTD vs No TD	−0.08	0.92	0.74 – 1.16	0.491
	PTD vs NDTD	0.13	1.14	0.91 – 1.43	0.250
**TPOAb**	PTD vs No TD	2.75	15.67	4.06 – 60.54	**<0.001**
	NDTD vs No TD	1.93	6.86	2.14 – 21.97	**0.001**
	PTD vs NDTD	0.83	2.28	0.71 – 7.34	0.165
**TgAb (TAT)**	PTD vs No TD	1.01	2.74	1.04 – 7.21	**0.041**
	NDTD vs No TD	0.78	2.18	0.95 – 5.03	0.066
	PTD vs NDTD	0.23	1.25	0.54 – 2.89	0.596

* Data are derived from generalised linear models (GLM) with Gamma distribution and log link, adjusted for age, sex and BMI. β represents the regression coefficient on the log scale and Exp(β) the exponentiated coefficient (ratio of means). Values are presented with 95% confidence intervals (CI). Reference group**:** No thyroid dysfunction (No TD).

† Statistical significance was defined as $p < 0.05$.

No TD = no thyroid dysfunction; PTD = pre-existing thyroid disease; NDTD = newly detected thyroid dysfunction.

Supplementary Table S6. Spearman correlations between thyroid markers and β-cell function in the post-COVID cohort

Variable	Outcome	n	ϱ	95% CI	*p*-value†
**TSH**	HOMA-B/HOMA-IR	35	-0.092	-0.418 – 0.263	0.610
**FT3**	HOMA-B/HOMA-IR	35	**0.421**	**0.083 – 0.672**	**0.018**
**FT4**	HOMA-B/HOMA-IR	35	-0.081	-0.409 – 0.269	0.640
**FT3/FT4 ratio**	HOMA-B/HOMA-IR	35	**0.382**	**0.041 – 0.643**	**0.031**
**TPOAb**	HOMA-B/HOMA-IR	35	-0.137	-0.487 – 0.252	0.410
**TAT (TgAb)**	HOMA-B/HOMA-IR	35	-0.061	-0.421 – 0.324	0.740

* Spearman correlation coefficients (ϱ) are presented with 95% confidence intervals (CI) and two-sided p-values. Confidence intervals were estimated using bootstrap resampling (1000 iterations).

† Statistical significance was defined as $p < 0.05$.

The associations between FT3 and β-cell compensatory capacity, as well as between the FT3/FT4 ratio and HOMA-B/HOMA-IR, are illustrated in Figure 3.

https://doi.org/10.3390/biomedicines14071420
